# High prevalence of porcine Hokovirus in German wild boar populations

**DOI:** 10.1186/1743-422X-7-171

**Published:** 2010-07-25

**Authors:** Cornelia Adlhoch, Marco Kaiser, Heinz Ellerbrok, Georg Pauli

**Affiliations:** 1 Robert Koch-Institut, Centre for Biological Security ZBS1, Nordufer 20, 13353 Berlin, Germany; 2 GenExpress GmbH, Eresburgstr. 22-23, 12103 Berlin, Germany

## Abstract

Porcine Hokovirus (PHoV) was recently discovered in Hong Kong. This new Parvovirus of pigs is closely related to the human Parvoviruses 4 and 5 (PARV4/5) and bovine Hokovirus (BHoV). So far, nothing is known about the presence and prevalence of PHoV in regions of the world other than Hong Kong. A study was initiated to investigate PHoV in German wild boars from five different geographical regions, using a newly established quantitative real-time PCR assay. Analysis of collected liver and serum samples revealed high overall prevalence (32.7%; 51/156) of PHoV in wild boars. The prevalence differed between the regions and increased with age. Two near full-length genomes and a large fragment for three additional isolates from different regions were sequenced and used for phylogenetic analysis. The German PHoV sequences from wild boars showed a close relationship with sequences of isolates from Hong Kong.

## Findings

A broad spectrum of parvoviruses is circulating worldwide in different species causing diseases in animals and humans. One of several novel animal parvoviruses is the porcine *Hokovirus (PHoV)*, a putative member of the genus *Parvovirus *within the family of *Parvoviridae*. This new parvovirus PHoV has been described in pigs from Hong Kong [1]. The non-enveloped parvovirus harbours a single-stranded DNA genome of approximately 5 kb. The genome has two open reading frames (ORFs) coding for non-structural and capsid proteins. Closely related human Parvoviruses PARV 4 and PARV5 were detected in various samples from healthy and diseased individuals [2-5].

Up to now no information is available about the presence of PHoV in other pig populations. This study was initiated to analyse PHoV in German wild boars. Wild boars in Germany are carrier of Hepatitis E virus (HEV) and it was of interest to analyse whether this species habours additional viruses with a zoonotic potential [6].

Liver, serum and bile samples from a total of 156 wild boars were tested for the presence of PHoV genomes. Samples (n = 127) were collected during the hunting season 2007/2008 at several sites in Germany. Collection points in the different federal states were described in a previous study on HEV [6]. Additional samples (9 wild boar livers) were collected at sites in the federal state of Hesse (HE) near Herleshausen/Werra, Bauhaus, Oberellen, Friedewald and Lengers between January and March 2008 and 20 wild boar serum samples were collected between November 2005 and January 2006 in the federal state of Baden Württemberg (BW) at different sites in the nature reserve Schönbuch which were nearly identical to the later sampling places in the hunting season 2007/2008. In general, sampling, age determination of animals, storing and handling of samples were carried out as published previously [6]. Briefly, liver samples (20 to 40 mg) were homogenized in 500 μl PBS using Precellys ceramic beads (diameter of 1.4 mm; Peqlab Biotechnology, Erlangen, Germany) and the FastPrep^® ^FP220A homogenizer (Qbiogene, MP Biomedicals, Solon, OH, USA). A volume of 200 μl of supernatant of the centrifugated homogenized liver, bile or serum samples was used for DNA extraction using the NucleoSpin^® ^Blood preparation kit (Macherey-Nagel, Düren, Germany). A quantitative real-time PCR (qPCR) assay using the PHoV_TM 5' nuclease probe (TaqMan^® ^probe) in combination with 3 primers PHoV_F/PHoV_R/HPV_R (Table 1; TIB MOLBIOL, Berlin, Germany) was applied in this study to determine the copy numbers of PHoV genomes. The assay was established to detect the newly described parvovirus PHoV and the human PARV4/PARV5 using primers binding within a conserved region for each virus. DNA samples in a volume of 2.5 μl were analysed using the following qPCR protocol in a final volume of 25 μl with 10xbuffer, 4 mM of MgCl_2_, dNTP 0.2 mM each, 0.3 μM of each primer, 0.1 μM of probe, ROX 0.1 μM and Platinum^® ^Taq 0.5 U. Platinum^® ^Taq DNA polymerase, MgCl_2 _and dNTPs were obtained from Invitrogen (Carlsbad, CA, USA) and water (Molecular Biology Grade) from Eppendorf (Hamburg, Germany). General reaction conditions for the real-time assay were 95°C for 10 min and 45 cycles with 95°C for 15 sec, 60°C for 35 sec. Reactions were run in an ABI GeneAmp^® ^7500 Detection System (Applied Biosystems, Foster City, CA, USA). Plasmid pHoko containing the 83 nucleotide (nt) amplification product from the isolate PHoV_BW2117 [GQ869539] was established. Insert was verified by sequencing and copy numbers in this preparation were calculated using standard methods. The plasmid was tenfold serially diluted in water containing γ-DNA (1 ng/μl) from 10^6 ^copies to 1 copy as standards for quantification of viral genomes. For qPCR each sample was analysed in duplicate. Copy numbers in samples were determined using a standard curve. The detection limit was estimated to be 10 copies of DNA per reaction. The β-Actin-qPCR assay was used as internal control [6]. The near full-length genomes were generated with PCR and nested PCR using several primer pairs in combination with primers for sequencing (Table 1) with the Platinum^® ^Taq DNA polymerase kit as described previously for HEV [6]. Sequence of amplicons was determined either directly using the PCR product or after cloning into vector pCR II TOPO (Invitrogen) by sequencing both strands with the Big Dye3.1 protocol using an automated sequencer (Genetic Analyzer 3130 xl, Applied Biosystems). Sequence data were analysed using ABI PRISM DNA Sequencing Analysis Software (Version 3.7, Applied Biosystems). Phylogenetic tree analysis was performed using MEGA 4.01 [7] program http://www.megasoftware.net and BLAST network program (National Center for Biotechnology Information, Bethesda, MD, USA).

**Table 1:** Overview of the analysed samples for PHoV.

Region	n_positive_/n_total_	<1 year	1-2 years	Adult	Unknown
**BW_2005**	2/20 (10.0%)	0/9	2/8	0/3	0/0
**BW_2007**	5/27 (18.5%)	0/10	2/7	0/4	3/6
**HE**	2/9 (22.2%)	1/3	1/4	0/2	0/0
**RP**	4/53 (7.5%)	1/27	0/14	3/11	0/1
**BB**	12/19 (63.2%)	4/9	3/3	3/3	2/4
**SA**	26/28 (92.9%)	9/9	10/10	7/9	0/0

**Total**	51/156 (32.7%)	15/67 (22.4%)	18/46 (39.1%)	13/32 (40.6%)	5/11 (45.5%)
**n C_T_<30**	17	7	9	1	0
%/Pos*	33.3	46.7	50.0	7.7	0
**%/Total**^†^	10.9	10.5	19.6	3.1	0

Number of PHoV positive and total tested animals with prevalences and virus load (C_T_-values) overall and divided by age groups and regions of sample collection.

*: estimated percentages per total samples tested positive; ^†^: estimated percentages per total samples tested; n: number of animals; BW: Baden Württemberg, HE: Hesse, RP: Rhineland Palatinate, BB: Brandenburg, SA: Saxony

The prevalence of PHoV in liver or serum samples of wild boars differed between sampling regions: While a low prevalence was seen in Rhineland Palatinate (RP), BW and HE, the samples collected in Saxony (SA) and Brandenburg (BB) showed high values (Table 2). The overall prevalence was 32.7% (51/156), 17 of 51 (33.3%) animals tested positive with C_T_-values lower than 30 indicating high copy numbers of more than 10^6 ^genome equivalents per mg of liver tissue, 16 of the 17 animals (94%) showing high copy numbers were below 2 years of age (7 animals <1 year, 9 animals 1-2 years). The analysis of the age distribution showed an increase in prevalence for animals older than 1 year, but the highest proportion of animals with high virus loads was seen in the group below 2 years of age (Table 1). Corresponding serum and liver tissue samples from six animals were tested in parallel showing comparable values for both compartments. Although it was shown previously that HEV was detected in high virus load in bile samples [6], quantification for PHoV in samples from three animals yielded virus loads that were up to 1000 times lower in bile samples than in the liver (data not shown). This result implicates, that PHoV has an organ tropism different from HEV. It can be assumed that a high virus load of PHoV in liver tissue and serum indicates an acute or persistent infection with a simultaneous viraemia.

**Table 2:** Used primers and probe for the quantitative analysis and generation of genome fragments of PHoV.

Primer name	Orientation 5'-3'
PHoV_F	gTT ggT CCT ggT AAT CCT YTg g
PHoV_R	TCg TAC CgT TCA TCg Tgg Tg
HPV_R	TgC gTA CCg TTC ATC ATg ATg TT
PHoV_TM	FAM-Agg gAC CAg Tgg ATg ARg CAg C-BBQ
PHoV_240F	CAC ACC TAC CTC gCC TAT AAg AAT C
PHoV_1273F	ggT AYT TTg CWg CHT ggg C
PHoV_1408R	CAA TTC ACR CAR CCR TAA gAW ggA
PHoV_1847F	CCg ATC TCC CCg TCT gCC
PHoV_2293F	CCg CAC TgA ggg CTA Cg
PHoV_2492F	ggT AAg MAA WCA TgW CWg CYg C
PHoV_2492R	gCR gCW gWC ATg WTT KCT TAC C
PHoV_4115F	ggg ARA ATT ATg TTY TKC CTC ART ATg g
PHoV_4395R	ATC WAC MCC TgT CAT RAT MgC
PHoV_5288R	CAC TgA TCA gAA ggM ACY TCR TAC AC

F: forward; R: reverse orientation; TM: TaqMan probe

**Figure 1:**
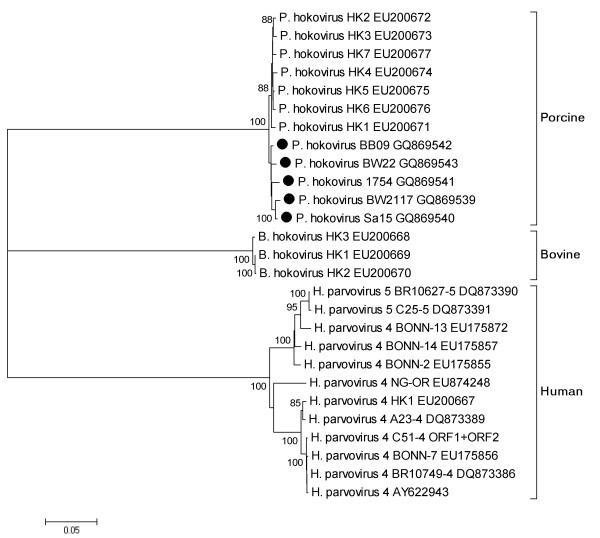
**Phylogenetic analysis**. A phylogenetic tree of new porcine, bovine and human parvoviruses was constructed using the neighbor joining method. Bootstrap values for the major branch points are given in percent. The trees were statistically evaluated in a bootstrap analysis with 1,000 replicates. The new German PHoV sequences are indicated with a dot.

In order to analyse the phylogenetic relationship between PHoV in Hong Kong and in Germany near full-length genome sequences with 4942 nt and 4944 nt were amplified from isolates BW2117 [GQ869539] and Sa15 [GQ869540], respectively. Additionally, discontinuous genome sequences of isolates from isolate BW22 [GQ869543], RP1754 [GQ869541] and BB09 [GQ869542] were generated with total sizes of 4564, 3027 and 3928 nt, respectively. All isolates were incomplete at the 3'-end of the VP1 and VP2 ORFs. The phylogenetic analysis showed that the PHoV isolates from German wild boars were closely related to Hong Kong isolates but formed a separate branch in the phylogenetic tree of all known porcine, bovine and human isolates from the GeneBank database (Figure 1). All German sequences were closely related to each other. The generated full-length sequences BW2117 and Sa15 differed in 44 of 4796 nucleotides (99.1% identity). A divergence of up to 40% was found to complete PARV4/5 sequences, and of 37% to bovine isolates. Compared to the isolates from Hong Kong a difference of 1.8-2.0% (Sa15) and 2.0-2.3% (BW2117) was seen for the German full-length sequences on nucleotide level.

Within NS1 7 (1.1%) unique amino acid exchanges were observed in the German isolates BW2117 and Sa15 in comparison to the Hong Kong isolates The phylogenetic analysis showed that within the ORFs coding for VP1 and VP2 proteins only one (VP1; 0.2%) and three (VP2; 0.3%) unique amino acid exchanges were found in the German isolates (BW2117 and Sa15) in comparison to the Hong Kong isolates.

In this study it was shown that the newly discovered PHoV is present in European wild boar populations. The virus was detectable in approximately every third animal tested. PHoV prevalence showed regional variation as determined in samples from animals collected in 5 geographic regions in Germany. The presence of high copy numbers of viral genomes in younger animals (≤ two years) points to an infection early in life. The increase of the prevalence in older animals supports the hypothesis of PHoV persistence in liver comparable to the situation observed for PARV4 and PARV5 infections in humans [8]. Therefore persistence might be a common feature for this new group of parvoviruses. So far, no clear disease has been linked to the infection or persistence of these new parvoviruses. The phylogenetic analysis showed a close relationship of the German PHoV sequences with the isolates from Hong Kong, although the European isolates clustered together in one separate branch. It can be speculated that the virus has been distributed through pigs that have been imported from Europe to Hong Kong.

Although the qPCR assay was established to detect all known isolates of the new Parvovirus group (PARV4, PARV5, PHoV and BHoV), only PHoV was found in the wild boar samples. The fact that approximately 600.000 wild boars are shot and consumed every year in Germany clearly highlights a potential route for a zoonotic transmission to humans. While the prevalence of PHoV in commercial pigs is yet unknown PHoV has been detected in a variety of porcine tissues with high virus load [1] indicating yet another potential risk of zoonotic transmission of PHoV to humans that urgently needs to be evaluated.

Acc. numbers of generated sequences:

[P._Hokovirus_BW2117: GQ869539; P._Hokovirus_Sa15: GQ869540; P._Hokovirus_1754: GQ869541; P._Hokovirus_BB09: GQ869542; P._Hokovirus_BW22: GQ869543]

## Abbreviations

BB: Brandenburg; SA: Saxony; RP: Rhineland Palatinate; BW: Baden Württemberg; HE: Hesse; qPCR: quantitative real-time PCR; PHoV: porcine Hokovirus; BHoV: Bovine Hokovirus; HEV: Hepatitis E virus

## Competing interests

The authors declare that they have no competing interests.

## Authors' contributions

CA: Study design, sampling, interpretation of the data and manuscript draft. MK: Sample analysis, phylogenetic analysis, interpretation of the data and manuscript draft. HE: Critical interpretation of the data and manuscript draft. GP: Study design, interpretation of the data and approval of the manuscript. All authors have read and approved the final manuscript.
